# Effects of N-Acetylcysteine in Ozone-Induced Chronic Obstructive Pulmonary Disease Model

**DOI:** 10.1371/journal.pone.0080782

**Published:** 2013-11-18

**Authors:** Feng Li, Cornelis Wiegman, Joanna M. Seiffert, Jie Zhu, Colin Clarke, Yan Chang, Pank Bhavsar, Ian Adcock, Junfeng Zhang, Xin Zhou, Kian Fan Chung

**Affiliations:** 1 Experimental Studies Unit, National Heart and Lung Institute, Imperial College London, London, United Kingdom; 2 Department of Respiratory Medicine, the Affiliated First People’s Hospital of Shanghai Jiao Tong University, Shanghai, P.R. China; 3 Department of Preventive Medicine, Keck School of Medicine, University of Southern California, Los Angeles, California, United States of America; University of California San Francisco, United States of America

## Abstract

**Introduction:**

Chronic exposure to high levels of ozone induces emphysema and chronic inflammation in mice. We determined the recovery from ozone-induced injury and whether an antioxidant, N-acetylcysteine (NAC), could prevent or reverse the lung damage.

**Methods:**

Mice were exposed to ozone (2.5 ppm, 3 hours/12 exposures, over 6 weeks) and studied 24 hours (24h) or 6 weeks (6W) later. Nac (100 mg/kg, intraperitoneally) was administered either before each exposure (preventive) or after completion of exposure (therapeutic) for 6 weeks.

**Results:**

After ozone exposure, there was an increase in functional residual capacity, total lung volume, and lung compliance, and a reduction in the ratio of forced expiratory volume at 25 and 50 milliseconds to forced vital capacity (FEV_25_/FVC, FEV_50_/FVC). Mean linear intercept (L_m_) and airway hyperresponsiveness (AHR) to acetylcholine increased, and remained unchanged at 6W after cessation of exposure. Preventive NAC reduced the number of BAL macrophages and airway smooth muscle (ASM) mass. Therapeutic NAC reversed AHR, and reduced ASM mass and apoptotic cells.

**Conclusion:**

Emphysema and lung function changes were irreversible up to 6W after cessation of ozone exposure, and were not reversed by NAC. The beneficial effects of therapeutic NAC may be restricted to the ASM.

## Introduction

 Chronic obstructive pulmonary disease (COPD) is a major cause of global morbidity and mortality, and is characterised by inflammation and fibrosis of the small airways and parenchymal destruction (emphysema), which ultimately contribute to irreversible airflow obstruction [1]. Cigarette smoking is the main etiological cause of COPD, but other factors including indoor and outdoor air pollution have also been associated with the development of COPD[2]. Oxidative stress, due to the increased exogenous oxidants from inhalation of cigarette smoke and environmental pollutants or endogenous oxidants generated from inflammatory cells, plays an important pathogenic role in COPD [3,4]. The development of a chronic exposure model to ozone in mice that results in chronic lung inflammation and emphysema also provides direct support for an important role for oxidative stress in COPD[5]. Oxidative stress mediates the recruitment of inflammatory cells, activates kinases such as mitogen-activated protein kinase and phospho-inositol-3-kinase and transcription factors such as NF-κB and AP-1 to up-regulate pro-inflammatory gene expression, that leads to a chronic inflammatory response [4,6] and the development of emphysema through the initiation of extracellular matrix proteolysis and endothelial and epithelial cell apoptosis [6,7]. In addition, oxidative stress on the lungs may induce airway smooth muscle hyperplasia and dysfunction, and contribute to ensuing bronchial hyperresponsiveness (BHR) (1,4,5). 

Currently, there are no effective treatments to reverse the process of COPD. N-acetylcysteine (NAC), an acetyl derivative of cysteine, which increases intracellular reduced glutathione (GSH) in the lungs and neutralizes oxidant species [8] has been used in the treatment of COPD as a mucolytic agent. NAC has been reported to reduce oxidative markers in COPD patients, but yielded mixed effects regarding any improvements in lung function [8,9]. In experimental studies, NAC attenuated elastase-induced emphysema in rats [10], but did not affect emphysema induced by cigarette smoke in mice [11]. The effect of NAC in a direct oxidant-induced model such as the chronic ozone exposure model is not known. 

In the present study, we examined the preventive and therapeutic effects of NAC on inflammation, pulmonary function, airway responsiveness, emphysema, and inflammatory gene expression in chronic ozone-induced murine model of COPD. In so doing, we also determined whether the ozone-induced lung changes could reverse spontaneously with time.

## Materials and Methods

 The experiments were performed within the legal framework of the United Kingdom under a Project License granted by the Home Office of Her Majesty's government. The researchers hold Personal Licenses provided by the Home Office of Her Majesty's government to perform the specific experiments described here.  

### A complete description is provided in the Online supplement in File S1

#### Ozone exposure and lung function

C57/BL6 mice (Harlan, UK) were exposed to ozone (3 hours; 2.5 parts per million) [5] (Figure S1). NAC (100mg/Kg, i.p) or PBS was administered one hour before ozone exposure twice a week for 6 weeks or after cessation of ozone exposure twice a week for 6 weeks. 

 At either the end of week 6 or 12, mice were anesthetized, tracheostomized and placed in a body plethysmograph (eSpira™ Forced Manoeuvers System, EMMS, Hants, UK). Functional residual capacity (FRC) was determined by Boyle’s law, and chord compliance (Cchord) was measured from the quasi-static pressure-volume manoeuvre. Inspiratory capacity (IC), total lung capacity (TLC), forced vital capacity (FVC) and the forced expiratory volume in first 25 and 50 milliseconds of exhalation (FEV_25_, FEV_50_) were recorded during fast-flow volume manoeuvre. 

 Airway responsiveness to acetylcholine (ACh) was measured in ventilated mice and transpulmonary pressure was assessed via an esophageal catheter. Pulmonary resistance (R_L_) was recorded in response to increasing concentrations of nebulised ACh. The concentration of ACh required to increase R_L_ by 150% from baseline was calculated (PC_150_).

 Following terminal anaesthesia, bronchoalveolar lavage (BAL) fluid was obtained. Total cell and differential cell counts from cytospins stained by Diff-Quick method were measured.

BAL malondialdehyde (MDA) was measured using a HPLC system with fluorescent detection (Waters, Milford, MA, USA). Serum 8-hydroxy-deoxyguanosine (8-OHdG), a major product of DNA oxidation, was quantified by solid phase extraction coupled with LC-MS/MS. 

### Lung morphometric analysis

 Five µm sections of paraffin-embedded left lung were stained with haematoxylin and eosin (H&E) or Masson’s trichrome. We examined 3-4 medium-sized bronchi within the range of 60 to 125 µm diameter. The severity of inflammatory response in peribronchial and perivascular in H&E sections was scored on a 0–3 scale: 0= no inflammatory response; 1=mild inflammation with foci of inflammatory cells in bronchial or vascular wall and in alveolar septa; 2=moderate inflammation with patchy inflammation or localized inflammation in walls of bronchi or blood vessels and alveolar septa, and less than one-third of lung cross-sectional area is involved; and 3=severe inflammation with diffuse inflammatory cells in walls of bronchi or blood vessels and alveoli septa; between one-third and two-thirds of the lung area is involved.

The mean linear intercept (L_m_), a measure of interalveolar septal wall distance, was determined and calculated by dividing the length of the line by the number of alveolar wall and grid line interception counted. 3-4 medium sized bronchial walls in Masson’s trichrome-stained sections were point-counted to assess morphological changes of airway epithelium, collagen deposition and airway smooth muscle (ASM) mass.

 Lung sections were incubated with peroxidase blocking solution (Dako, Cambridge, UK) and rabbit anti-apoptosis protease activating factor-1 (APA-1) primary antibody, polyclonal goat anti-rabbit horseradish peroxidase (HRP)-conjugated secondary antibody followed by diaminobenzidine (DAB) liquid. The immunostaining intensity for APA-1 in the airway epithelium was scored on 0-3 scale. The number of APA-1 positive cell per field was also counted.

### Reverse transcription and real-time PCR

Total RNA was extracted from lungs using an RNeasy Mini Kit (Qiagen, West Sussex, UK) and then was reverse-transcribed to cDNA. Real-time PCR analyses were performed to determine mRNA levels of IL-1β, Caspse-3, TGF-β, MMP-9, SOD-2 and HO-1 using Rotor-Gene 3000 (Corbett Research, Sydney, Australia) and QuantiTect SYBR Green PCR Master Mix Reagent (Qiagen). Relative abundance of gene expression was normalized to 18S rRNA expression.

### Data analysis

 Data are presented as mean ± S.E.M. Two-way analysis of variance was performed for comparisons of %change in RL between individual groups within Groups 1, 2 and 3 and Groups 4, 5 and 6. One-way analysis of variance with LSD post-test (equal variance) or Dunnett’s T3 post-test (unequal variance) was performed for comparison between multiple groups within Groups 1, 2 and 3 and Groups 4, 5 and 6. Unpaired t-test or Mann-Whitney test was carried out for comparison between ozone group (Group 2) and ozone recovery group (Group 5). A p value of <0.05 was considered significant.

## Results

### Pulmonary function measurements

**Figure 1:**
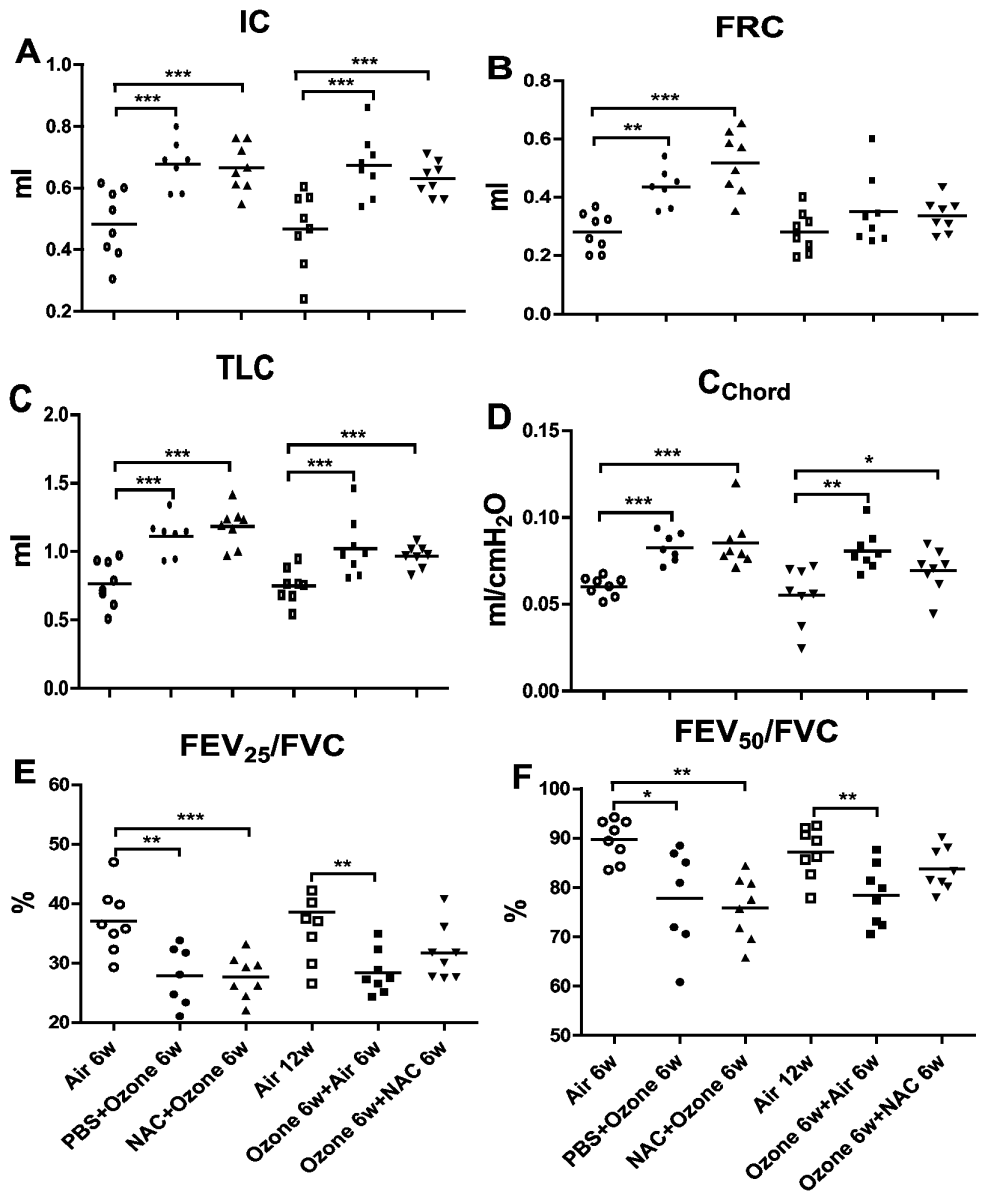
Lung mechanics. Individual and mean values of inspiratory capacity (IC)(Panel **A**), functional residual capacity (FRC) (Panel **B**), total lung capacity (TLC) (Panel **C**), chord compliance (C_chord_) (Panel **D**), and percentage of forced expiratory volume (FEV) in first 25 and 50 ms of fast expiration (FEV_25_ and FEV_50_) of forced vital capacity (FVC)(Panels E,F). ^*^compared with air control, ^*^P<0.05, ^**^P<0.01, ^***^P<0.001.

There were significant increases in lung volume parameters (IC, FRC and TLC) and compliance (C_chord_) and decreases in airflow during expiration (FEV_25_/FVC, FEV_50_/FVC) in PBS-pretreated ozone-exposed mice compared to air-exposed group (Figure 1). Six weeks after cessation of exposure, the increase in IC, TLC, C_chord_ but not in FRC remained elevated and the decrease in FEV_25_/FVC and FEV_50_/FVC also were unchanged. Preventive and therapeutic NAC treatment did not have beneficial effect on any of these parameters. 

### Airway responsiveness

**Figure 2:**
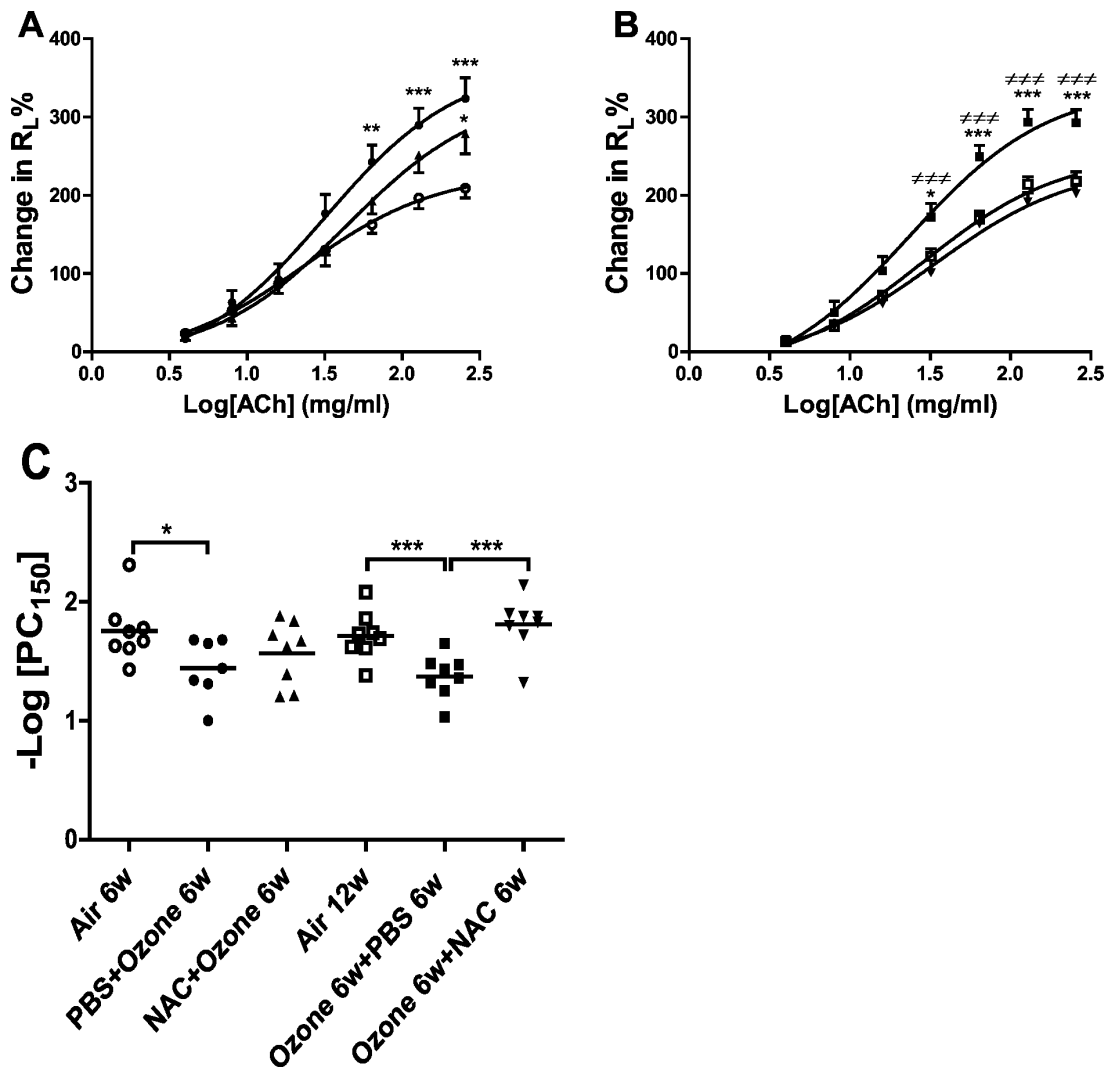
Airway hyperresponsiveness. Mean percentage increase in lung resistance (R_L_) to increasing concentrations of acetylcholine is shown in **Panel** **A**.. Three groups of mice were studied: air-exposed mice (n=8), PBS-pretreated ozone-exposed mice (n=7), NAC-pretreated ozone-exposed mice (n=8). ^*^P<0.05, ^**^P<0.01, ^***^P<0.001 compared with air control. Data is expressed as means ±S.E.M. ○: Air exposure 6w,●: ozone-exposure 6w,▲:ozone-exposure and NAC-treatment 6w. Panel **B**. Mean percentage increase in lung resistance to increasing concentrations of acetylcholine, ^*^P<0.05, ^**^P<0.01, ^***^P<0.001 compared with air control; ^≠^P<0.05, ^≠≠^P<0.01, ^≠≠≠^P<0.001 compared with ozone-exposed NAC treated group. Data is expressed as means ±S.E.M. □:Air exposure 12w, ■: ozone-exposure 6w and then PBS-treatment 6w,▼: ozone-exposure 6w and then NAC-treatment 6w. Panel **C**. Individual and mean –log PC_150_ of the six experimental groups. ^*^P<0.05, ^**^P<0.01.

PBS-pretreated ozone-exposed mice demonstrated a significant increase in airway responsiveness to ACh compared to air control mice (Figure 2C). Preventive NAC treatment did not alter the airway hyperresponsiveness seen in ozone-exposed mice (Figure 2A). After 6 weeks of recovery, ozone-exposed PBS-treated mice still demonstrated an increased airway responsiveness to ACh (P<0.001). Therapeutic intervention with NAC reversed the airway responsiveness of ozone-exposed mice compared to ozone-exposed PBS-treated mice (P<0.001; Figure 2B).

### BAL cells

**Figure 3:**
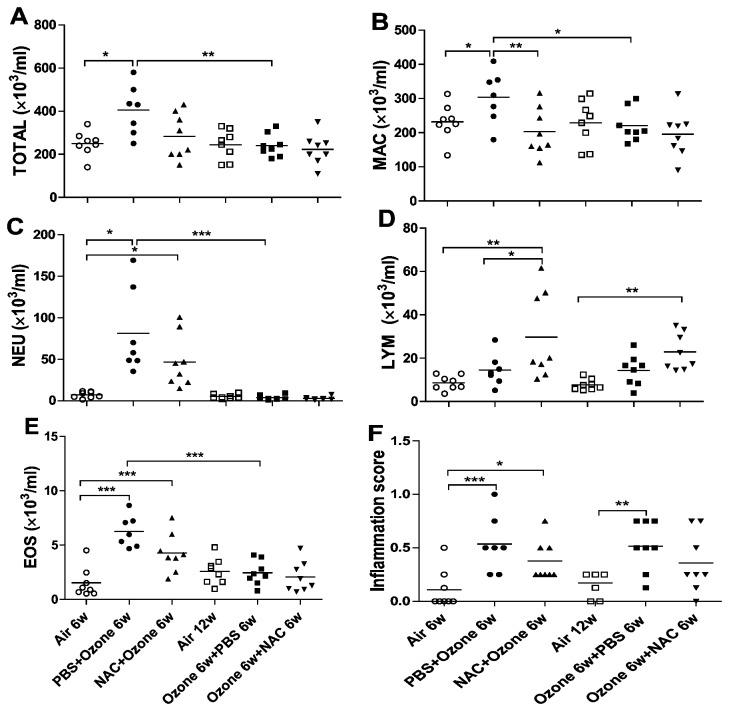
Bronchoalveolar lavage cells. Individual and mean numbers of total cells (TOTAL) (Panel **A**), macrophages (MAC) (Panel **B**), neutrophils (NEU) (Panel **C**), lymphocytes (LYM) (Panel **D**), and eosinophils (EOS) (Panel **E**) in bronchoalveolar lavage (BAL) fluid for the 6 experimental groups. Panel **F**. Individual and mean inflammation scores in the airways and lungs. ^*^P<0.05, ^**^P<0.01, ^***^ P<0.001.

PBS-pretreated ozone-exposed mice demonstrated an increase in total cell counts in BAL fluid (P<0.01), as reflected by increased macrophages (P<0.05), neutrophils (P<0.001) and eosinophils (P<0.001) compared to air-exposed mice (Figure 3A-E). After 6 weeks cessation of ozone exposure, there was a decrease in total cell counts including macrophage, neutrophil and eosinophil in BAL fluid. Preventive NAC treatment decreased macrophage numbers (P<0.01) and increased lymphocyte numbers (p<0.05) compared to PBS-pretreated ozone-exposed mice; therapeutic treatment with NAC in ozone-exposed mice did not affect total or constituent cell counts.. 

### Lung inflammation scores

Ozone exposure in PBS-pretreated mice caused an increase in inflammation scores with peribronchial and perivascular inflammatory cell infiltrates in lung sections compared to air-exposed mice (P<0.001, Figure 3F). This inflammatory response persisted up to 6 weeks after cessation of exposure to ozone. Preventive treatment with NAC did not lead to a reduction in lung inflammation scores. Similar findings were observed with therapeutic NAC. 

### BAL MDA levels

**Figure 4:**
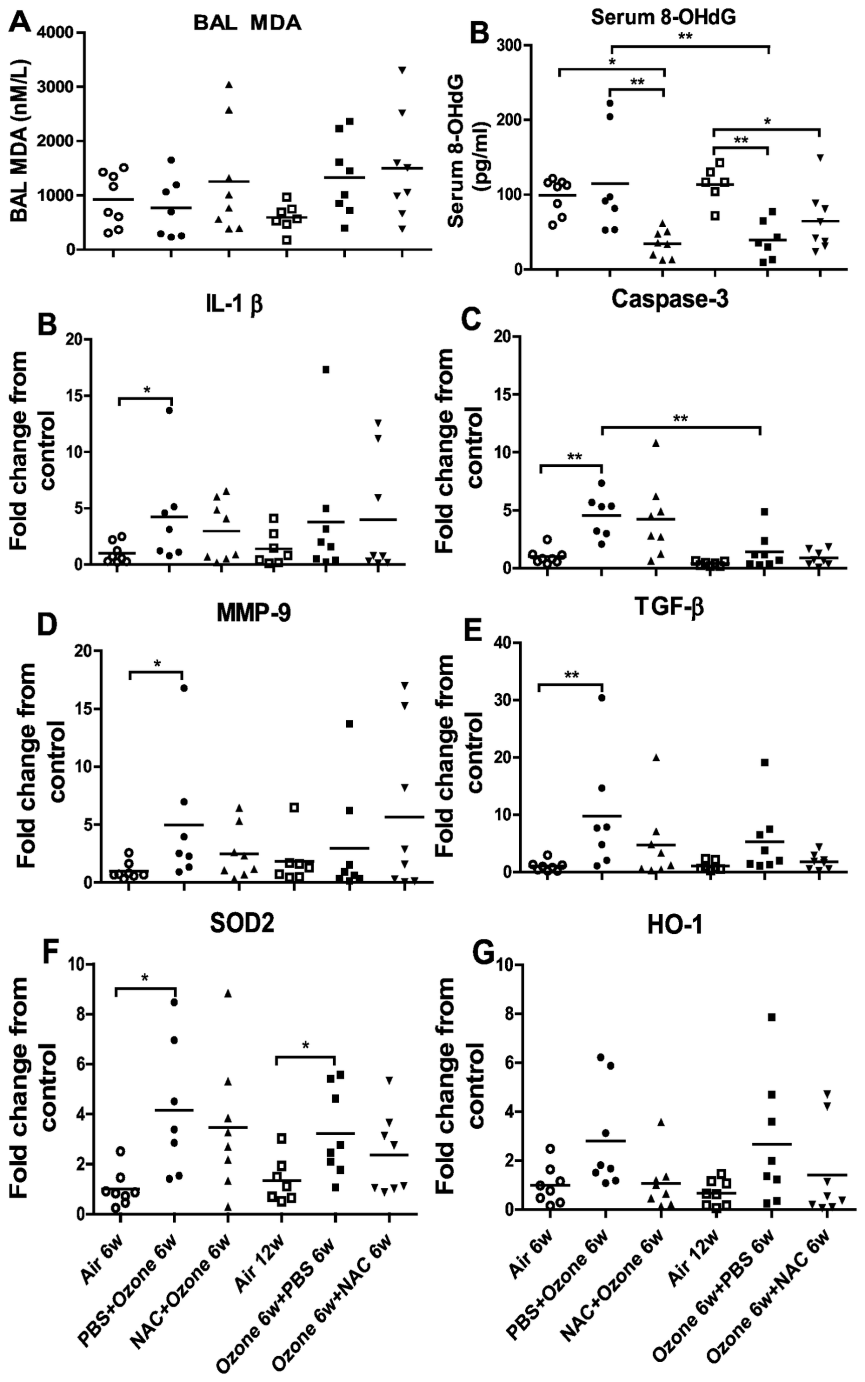
Bronchoalveolar lavage fluid and plasma mediators. Individual and mean concentrations of malonaldehyde in bronchoalveolar lavage fluid (Panel **A**). Plasma levels of 8-hydroxy-deguanosine is shown in **Panel** **B**. Individual and mean expression of IL-1β (Panel **C**), caspase-3 (Panel **D**), MMP-9 (Panel **E**), TGF-β (Panel **F**), SOD2 (Panel **G**) and HO-1(Panel **H**) in lung tissue measured by quantitative RT-PCR. ^*^P<0.05, ^**^P<0.01, ^***^ P<0.001.

Ozone exposure did not lead to an increase in MDA concentration at 6 weeks, but after 6 weeks of cessation levels increased significant, indicating a delayed oxidant response (Figure 4A). Preventive NAC did not affect MDA concentrations. Therapeutic NAC did not decrease the raised MDA levels observed after 6 weeks cessation of exposure.

### Plasma 8-hydroxy-deguanosine (8OH-*d*G)

Ozone exposure of PBS-pretreated mice did not increase the levels of 8-OHdG compared to air-exposed mice (Figure 4B). Stopping ozone exposure for 6 weeks decreased the concentration of 8-OHdG in ozone-exposed PBS-treated mice (P<0.01) and air control mice(P<0.01). Preventive treatment with NAC reduced the levels of 8-OHdG (P<0.01), while therapeutic NAC intervention had no effect.

### Mean linear intercept, L_m_

**Figure 5:**
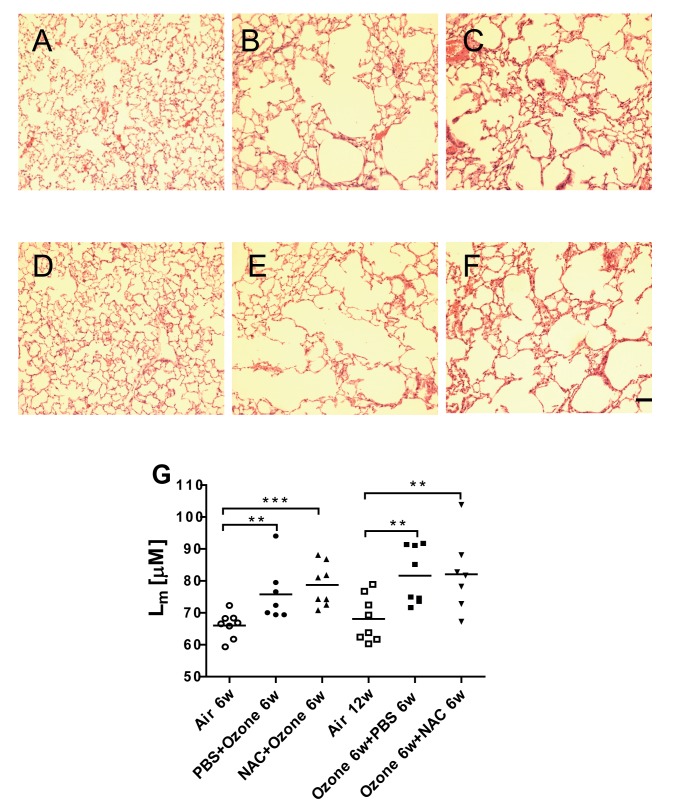
Evidence of emphysema. Representative photomicrographs of lung alveolar spaces in haematoxylin-eosin-stained sections of air-exposed mice (Panel **A**), PBS-pretreated ozone-exposed mice (Panel **B**), NAC-pretreated ozone-exposed mice (Panel **C**), air-exposed mice (Panel **D**), ozone-exposed PBS-treated mice (Panel **E**) ozone-exposed NAC-treated mice (Panel **F**). Scale bar = 40µm. Panel **G**. Individual and mean values of mean linear intercept, L_m_, in the lung sections from the 6 experimental groups. ^*^P<0.05, ^**^P<0.01, ^***^ P<0.001.

Figure 5 A-F shows alveolar enlargement at 6 weeks of ozone exposure, that persisted in mice after 6 weeks of cessation of exposure. This was reflected in an increase in L_m_ after 6 weeks of exposure (P<0.01) and also after 6 weeks of cessation of exposure (P<0.01; Figure 5G). Neither preventive nor therapeutic NAC inhibited the ozone-induced increase in L_m_. 

### Bronchial wall changes

**Figure 6:**
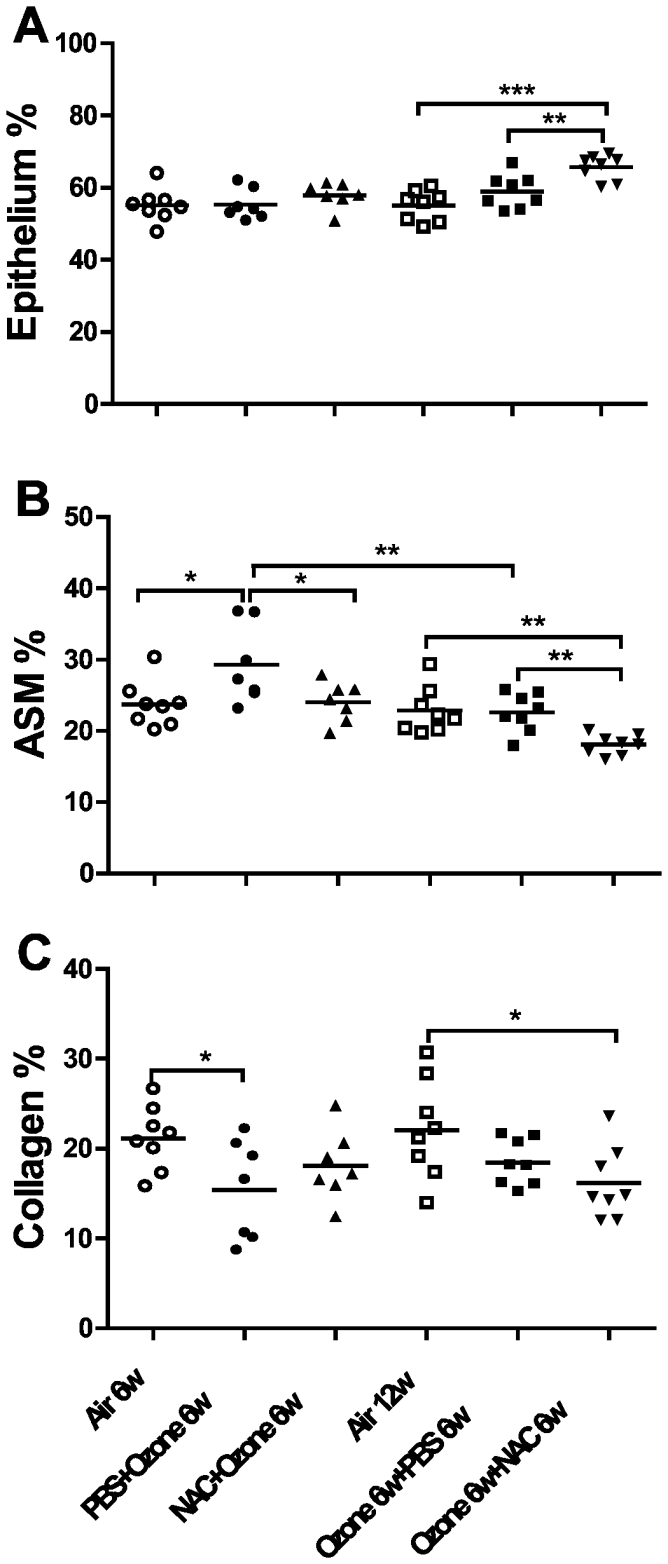
Changes in airway wall. Individual and mean values of relative proportion of epithelium (Panel **A**), airway smooth muscle (ASM) (Panel **B**) and collagen (Panel **C**) in the bronchial wall measured by point-counting of Masson trichrome stained sections. ^*^P<0.05, ^**^P<0.01, ^***^ P<0.001.

 Ozone exposure induced an increase in the proportion of ASM (P<0.05, Figure 6B), associated with decreased proportion of collagen (P<0.05, Figure 6C) in PBS-pretreated mice exposed to ozone. There was recovery of the increase in ASM cells after stopping exposure for 6 weeks (P<0.01). Both preventive and therapeutic treatments with NAC significantly reduced the proportion of ASM in the bronchial wall compared to PBS-pretreated ozone-exposed mice (P<0.05 and <0.001, respectively). However, NAC did not have an effect on the ozone-induced reduction in collagen. Interestingly, therapeutic NAC increased the epithelial area following ozone exposure (Figure 6A). 

### APA-1

**Figure 7:**
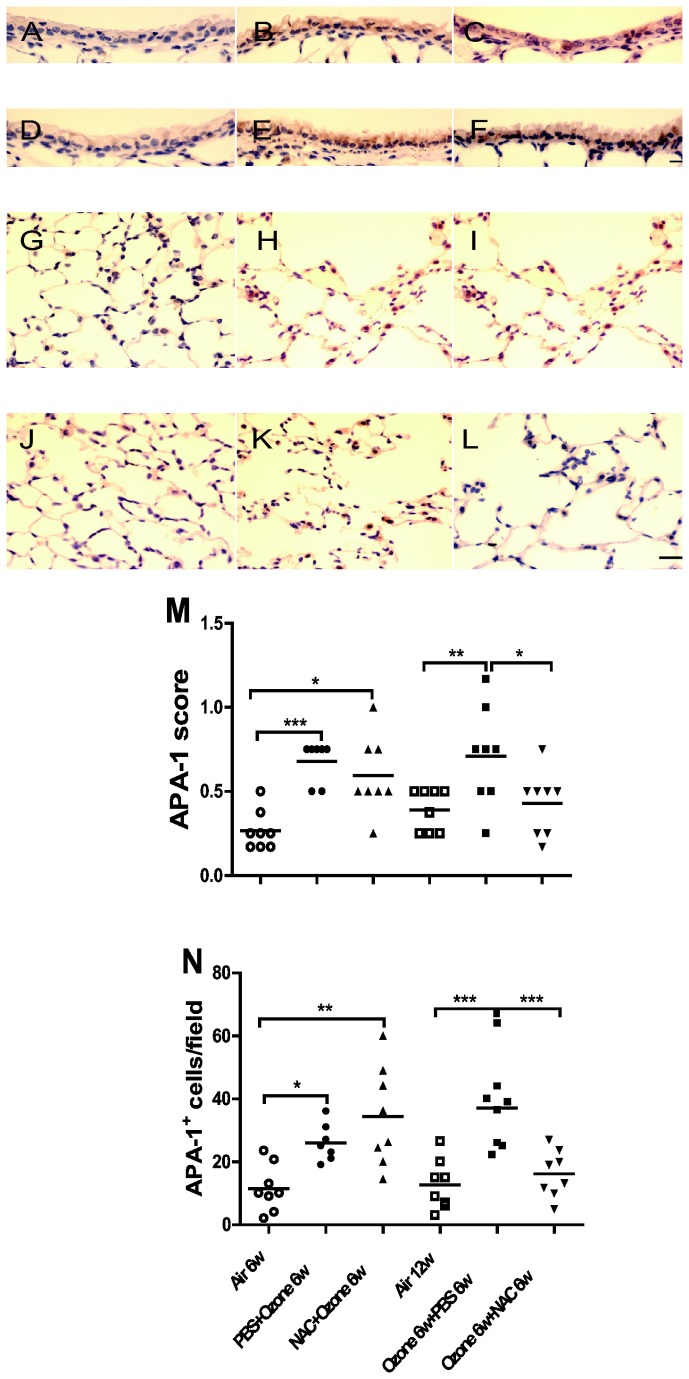
Apoptotic cells. Immunohistochemical analysis of the expression of apoptosis protease activating factor-1 (APA-1) in airway epithelium and parenchyma of air-exposed mice (Panel **A**, **G** ), PBS-pretreated ozone-exposed mice (Panel **B**,**H** ), NAC-pretreated ozone-exposed mice (Panel **C**, **I** ), air-exposed mice (Panel **D**, **J**), ozone-exposed PBS-treated mice (Panel **E**, **K**) ozone-exposed NAC-treated mice (Panel F, L). Scale bar: 10µm for airway epithelial pictures and 20μm for lung parenchymal pictures. Panel **M**. Individual and mean immunostaining scores of staining for APA in airway epithelial cells from the 6 experimental groups. Panel **N**. Individual and mean score values of APA-1 positive cells counted in the lung parenchyma. ^*^P<0.05, ^**^P<0.01, ^***^ P<0.001.

Ozone-exposed mice showed increases in APA-1 expression in the airway epithelium (P<0.001) and APA-1-positive cells (P<0.05, Figure 7), which persisted at 6 weeks after cessation of exposure. Preventive NAC treatment did not reduce the increased expression intensity or positive cell number of APA-1 induced by ozone. Therapeutic NAC treatment reversed the increase of APA-1 in the airway epithelium and parenchyma compared to ozone-exposed PBS-treated mice (P<0.05 and P<0.001, respectively).

### Lung gene expression

 Ozone exposure in PBS-pretreated mice evoked an increased mRNA expression of IL-1β, caspase-3, MMP-9, TGF-β and SOD2 in the lung tissue (Figure 4 B-G). Apart from caspase-3 expression which fell, there was no change in the expression of IL-1β, MMP-9, TGF-β, SOD2 and HO-1 after 6 weeks of cessation of exposure. Both preventive and therapeutic NAC treatment did not affect the mRNA levels of IL-1β, caspase-3, MMP-9, TGF-β, SOD2 and HO-1 in ozone-exposed mice compared to PBS-pretreated ozone-exposed mice 

## Discussion

 We demonstrated that lung inflammation, emphysema, abnormal lung function and AHR were induced by 6 weeks of ozone exposure and that these persisted up to 6 weeks after cessation of exposure. The increased numbers of neutrophils, eosinophils and macrophages in BAL fluid and the increased airway smooth muscle mass were reversed within 6 weeks. Therapeutic but not preventive NAC reduced AHR together with a reversal of increased airway smooth muscle mass and cellular apoptosis. Thus, while there was no recovery of ozone-induced lung inflammation and emphysema, there were therapeutic effects of NAC on AHR. The overall preventive and therapeutic effects of NAC are summarised in Table 1.

**Table 1:** Preventive and therapeutic effects of N-acetylcysteine on chronic ozone effects.

**Parameter**	**Preventive NAC**	**Therapeutic NAC**
IC, FRC, TLC, C_chord_	NE	NE
FEV25/FVC, FEV**^50^**/FVC	NE	↓
Airway hyperresponsiveness	NE	Reverses AHR
Bronchoalveolar lavage cells	↓macrophage ↑lymphocyte	NE
Lung inflammation score	NE	NE
Mean linear intercept	NE	NE
Airway smooth muscle mass	↓	↓
Epithelium	NE	↑
Collagen	NE	NE
APA-1 cells	NE	↓
IL-1β, Caspase 3, MMP-9, TGFβ mRNA expression	NE	NE
SOD2 mRNA expression	NE	NE
HO-1 mRNA expression	NE	NE
BAL malondialdehyde levels	NE	NE
Plasma 8-hydroxy-deoxyguanosine	↓	NE

Abbreviations: APA : apoptosis protease activating factor-1 ; C_chord_ chord compliance; FEV: forced expiratory volume; FRC: functional residual capacity; FVC: forced vital capacity; FEV_25_ and FEV_50:_ percentage of FEV in first 25 and 50 msec of fast expiration of FVC; HO-1 : haemoxygenase-1; IC: inspiratory capacity; IL-1β: interleukin-1β; MMP-9 : macrophage metalloproteinase 9; NE: no effect; SOD2 : superoxide dismutase 2 ; TGFβ : transforming growth factor β ; TLC: total lung capacity.

Chronic ozone exposure caused lung inflammation as measured by increased numbers of BAL macrophages, neutrophils and eosinophils, and by inflammatory cells in lungs. BAL inflammation disappeared 6 weeks after cessation of exposure, while lung inflammation scores remained elevated at 6 weeks. BAL inflammatory cells may be the reflection of the transient effect of the final exposure to ozone, while the persistence of the peribronchial and perivascular inflammation in the lung tissue may represent a more chronic process. On the other hand, gene expression of the pro-inflammatory cytokine, IL-1β, remained elevated, and were unaffected by NAC. IL-13, KC and IFNγ mRNA expression are also increased in the model, as previously shown [5]. Increased lung TGF-β mRNA that may contribute to airway wall remodelling and small airway fibrosis [12] was also observed. 

The increase in mean linear intercept after chronic ozone exposure indicates an increase in alveolar size and is in line with the increase in the lung volumes IC, FRC and TLC, with airflow obstruction as indicated by a reduction in the ratios of FEV_25_/FVC and FEV_50_/FVC. In addition, there was loss of lung elastic recoil as noted by increased lung compliance. The increase in alveolar size is most likely the result of alveolar damage and loss of alveoli as shown on the histological lung sections in Figure 5. The lung function changes observed on cessation of ozone exposure are concordant with previous studies of cigarette smoke exposure. In a smoke-induced emphysema model in A/J mice, inflammation and emphysema persisted after smoke cessation [11], while in another, inflammatory cells and cytokines were decreased in BAL fluid, but lung tissue inflammation and alveolar enlargement remained unchanged after smoke cessation [13]. We found up-regulation of lung MMP-9 mRNA which can recruit inflammatory cells and degrade anti-trypsin, resulting in unchecked protease activity and tissue damage including airspace enlargement [14]. We have previously shown an increase in the protein expression of another metalloproteinase, MMP-12, in our model [5]. Both MMP-9 and MMP-12 are likely to be involved in ozone-induced emphysema. Apoptosis of structural cells has also been proposed as a mechanism of emphysema [15]. Markers of apoptosis, caspase-3 gene expression and APA-1 protein expression in the lung tissue, were increased after chronic ozone exposure. However, caspase-3, not APA-1, receded spontaneously after 6 weeks of cessation of ozone exposure, indicating that some upregulated genes can revert with time to pre-exposure levels. 

 AHR is another feature of COPD, occurring in a large proportion of COPD patients [16,17]. A single ozone exposure induces neutrophilic inflammation and transient AHR [18,19] and the current study shows that chronic ozone exposure can also induce sustained AHR. Associated with this, the proportion of airway smooth muscle increased in ozone-exposed mice but this returned to baseline values by 6 weeks. Increased airway smooth muscle mass, increased airway smooth muscle contractility and activation of p38MAPK-HSP27 pathway are among the potential mechanisms for ozone-induced AHR [20]. Our study also demonstrated that preventive NAC did not prevent ozone-induced AHR, but it reversed AHR during ozone cessation period, which was associated with spontaneous recovery of airway smooth muscle mass to baseline. NAC has been shown to partly reduce LPS-induced AHR in mice [21] and AHR induced by mycotoxin in an allergic asthma model [22]. Thus, there is a reversible AHR component only with therapeutic NAC, while the airway smooth muscle changes reverted spontaneously.

 Chronic ozone exposure resulted in an increase in BAL MDA levels at six weeks after cessation of exposure, indicating a delayed oxidative stress signal in the lungs. The expression of the antioxidant superoxide dismutase 2 (SOD2) was enhanced in response to ozone exposure. Both preventive and therapeutic NAC had no effect on MDA levels in BAL of ozone-exposed mice; however, preventive treatment with NAC led to a reduction in plasma levels of the oxidative marker, 8-hydroxy-deoxyguanosine. Ozone exposure itself could induce some protective mechanism such as the promotion of CX3CR1-dependent maturation of resident lung macrophages to limit oxidative stress and inflammation [23]. NAC also did not alterthe gene expression levels of SOD2 and HO-1. Overall, the evidence we have would indicate that NAC was not having an antioxidant effect. However, the possibility still remains that the antioxidant effect was occurring intracellularly and was not reflected in the levels of MDA or 8-hydroxy-deoxy-guanosine. However, when given preventively, there was some evidence of a systemic antioxidant effect. 

Both preventive and therapeutic NAC administration did not affect or reverse the development of ozone-induced emphysema and this was associated with the failure to inhibit caspase-3 or MMP-9 expression. However, therapeutic NAC led to the inhibition of APA-1 expression and of APA-1 positive cells. Previous studies have reported varying effects of NAC in emphysema models. Thus, emphysema severity in cigarette smoke-exposed mice was not improved by treatment with NAC [11], while other studies in mouse or rat models of emphysema including those induced by cigarette smoke exposure showed prevention or amelioration of emphysema by preventive NAC [24–26]. It is likely that our model represents an advanced state of emphysema.

There is some discordance in terms of the differential effects of preventive or therapeutic administration of NAC (Table 1). For example, preventive NAC reduced BAL macrophage numbers while therapeutic NAC reversed AHR and the increase in airway smooth muscle mass, and the apoptotic scores. There is no rational explanation for these differential effects. One possibility is that the processes underlying the effects are more readily reversed than preventable. The bronchial hyperresponsiveness is likely to be related to the changes in airway smooth muscle. The lack of effect of preventive NAC could represent a poor effect of NAC in preventing oxidative stress induced by ozone, while therapeutic NAC could be more effective at later stages of oxidative stress likely to be caused by endogenous processes such as the activation of inflammation.

In COPD, smoking cessation is the only effective intervention that could slow the accelerated decline in pulmonary function [27] and improve AHR [28]. However, airway inflammation and emphysema persist or even progress after 4 years of smoking abstinence [29]. Some of the features of the chronic ozone model are similar to these clinical observations. In this mouse ozone model, we also demonstrate that there is no substantial recovery of the damage inflicted by a chronic oxidant stress. NAC administered therapeutically also did not affect emphysema and the inflammatory process, but improved AHR and ASM mass which may be the basis for potential clinical benefits of NAC in COPD. 

## Supporting Information

Figure S1 **Protocol used in the six experimental groups.**
(EPS)

File S1 **Contains full description of Methods and legend to Figure S1.**
(DOCX)
