# Supplementary material for: Effects of N-Acetylcysteine in Ozone-Induced Chronic Obstructive Pulmonary Disease Model
Source: PLoS One. 2013 Nov 18;8(11):e80782. doi: 10.1371/journal.pone.0080782 (PMC3832609; doi:10.1371/journal.pone.0080782)
Supplement: File S1 — Contains full description of Methods and legend to Figure S1. (DOCX) [file pone.0080782.s002.docx]

**Effects of N-acetylcysteine in ozone-induced murine model of chronic obstructive pulmonary disease**

Feng Li, Cornelis Wiegman, Joanna M Seiffert, Jie Zhu, Colin Clarke, Yan Chang, Pankaj Bhavsar, Ian Adcock, Junfeng Zhang, Xin Zhou, Kian Fan Chung

**Online Data supplement**

*Material and methods:*

**Materials and methods**

The experiments were performed within the legal framework of the United Kingdom under a Project License granted by the Home Office of Her Majesty's government. The researchers hold Personal Licenses provided by the Home Office of Her Majesty's government to perform the specific experiments described here.

*Mice and ozone exposure*

Pathogen-free, 10-12 week old male C57/BL6 mice (Harlan, UK) were housed within ‘maximiser’ filter-topped cages (Maximiser, Theseus caging system, Hazelton, PA, USA) in the Biosciences facilities of Imperial College, under controlled temperature (20°C) and humidity (40–60%), in a 12-h light/ 12-h dark cycle with food and water supplied *ad libitum*. The protocols and procedures used in the study were approved by the Animal Ethics Committee of Imperial College in compliance with UK Home Office regulations.

Mice were exposed to ozone produced from a generator (Model 500 Sander Ozoniser, Germany), mixed with air for 3 hours at a concentration 2.5 parts per million (ppm) in a sealed Perspex container(S1). Ozone concentration was continuously monitored using an ozone probe (ATi Technologies, Ashton-U-Lyne, UK). Ozone exposure was carried out in 4 groups (Fig E1) :

(1) two exposures (every 3 days) per week plus twice phosphate buffered saline (PBS, Sigma-Aldrich, Poole, UK) (0.3ml) treatment one hour before ozone exposure per week for 6 weeks,

(2) two exposures (every 3 days) per week plus twice NAC (100mg/Kg,i.p, dissolved in PBS,Sigma-Aldrich, Poole, UK) treatment one hour before ozone exposure per week for 6 weeks,

(3) two exposures per week over 6 weeks and then discontinued and treated with twice PBS (0.3ml) per week for 6 weeks,

(4) two exposures per week over 6 weeks and then discontinued and treated with twice NAC(100mg/Kg,i.p) per week for 6 weeks.

Two groups of control mice were exposed to air over the same period.

*Pulmonary function measurements*

At either the end of week 6 or of week 12, mice were anesthetized with an intraperitoneal injection of anesthetic solution containing ketamine (100 mg/Kg, Bayer healthcare, Kiel, Germany) and xylazine (10mg/Kg, Fort Dodge Animal health, Southampton, UK) while maintaining spontaneous breathing. Mice were tracheostomized and placed in a body plethysmograph (eSpira™ Forced Manoeuvres System, EMMS, Hants, UK). Three semiautomatic maneuvers were performed with the system by the use of negative pressures generated in the plethysmograph to generate quasi-static pressure-volume and fast flow-volume manoeuvre (S2). Functional residual capacity (FRC) was determined by Boyle’s law, and the chord compliance (C_chord_), a measure of the compliance on expiration between 0-10 cmH_2_O of pressure, was determined from the quasistatic pressure-volume manoeuvre. From quasi-static pressure-volume loops, inspiratory capacity (IC), total lung capacity (TLC), forced vital capacity (FVC) and the forced expiratory volume in first 25 and 50 milliseconds of exhalation (FEV_25_, FEV_50_) were recorded during fast flow volume manoeuvre and calculated based on the measurement of FRC.

*Measurement of airway responsiveness*

After the body plethysmographic measurements, mice were transferred to Resistance and Compliance plethysmograph (EMMS) for measuring airway responsiveness to acetylcholine (ACh) (Sigma, Dorset, UK) as described previously(S3, S4). Mice were ventilated with MiniVent type 845 (Hugo Sach Electronic, Germany) at a rate of 200 breaths/min and tidal volume of 250μl, and were monitored with a pneumotachograph connected to a transducer. Transpulmonary pressure was assessed via an esophageal catheter. Instantaneous calculation of pulmonary resistance (R_L_) was obtained. Increasing concentrations of ACh (4-256 mg/ml) were administered with an Aeroneb^®^ Lab Micropump Nebulizer (EMMS, Hants, UK), and R_L_ was recorded for a 3-min period following each concentration. R_L_ after each concentration was expressed as percentage change from baseline R_L_ measured following nebulized PBS solution. The concentration of ACh required to increase R_L_ by 150% from baseline was calculated (PC_150_) and –log PC_150_ was taken as a measure of airway responsiveness.

*Bronchoalveolar lavage*

Following terminal anaesthesia with pentobarbitone, mice were lavaged with 2 ml of PBS via a 1mm diameter endotracheal tube, and bronchoalveolar lavage (BAL) fluid was retrieved. The retrieved lavage aliquots were pooled and centrifuged at 4ºC, 250g, for 10 min and the supernatant was collected and stored at -80ºC. The cell pellet was re-suspended in PBS and counted using a hemocytometer. Total cell counts and differential cell counts from cytospin preparations stained by Diff-Quick method (Gentaur, Kampenhout, Belgium) were measured. At least 500 cells were counted per mouse and identified as macrophages, neutrophils, lymphocytes and eosinophils according to standard morphology under x400 magnification.

*BAL Malondialdehyde* *(MDA)*

MDA was measured using a HPLC system with fluorescent detection (Waters, Milford, MA, USA). A 20 µl aliquot sample was added into a mixture of 500 µl phosphoric acid (440 mM) and 250 µl thiobarbituric acid (TBA, 42 mM). After 1-hour reaction with TBA at 80°C in an oven, a 20 µl aliquot of this final solution was injected into the HPLC system with fluorescence detector set at 532 nm for the excitation wavelength and 553 nm for the emission wavelength. A Nova-Pak C18 column (Waters, Milford, MA, USA) was used with a mobile phase that was composed of 40% methanol and 60% water containing 50mM KH2PO4 (pH=6.8) at a flow rate of 0.8 ml/min. The detection limit, extraction recovery and analytical precision of this method were 1.8 nM, 75.9%, and 2.2% (measured as RSD from 8 replicate injections), respectively.

*Serum 8-hydroxy-deoxyguanosine (8-OHdG)*

Blood was taken from the left heart through a syringe with 25G needle and collected into tubes, then left to clot at 4ºC followed by centrifugation at 4ºC, 2400g, for 10 min. The serum was collected and stored at -80 ºC. Serum 8-OHdG was quantified by solid phase extraction (SPE) coupled with LC-MS/MS. Serum sample was spiked with 30 µL of 0.01 ng/µl of ^15^N_5_-8-OHdG (Cambridge Isotope Laboratories, Andover, MA , USA) and diluted with 1 mL of 0.1M KH2PO4 (pH=6.0). After vortexing and centrifugation, the sample was purified using Waters Sep-Pak C18 cartridge (Waters, Milford, MA, USA). The cartridge was prewashed with 3 mL of methanol and 3 mL of water; then the sample was loaded and the cartridge was washed with 1 mL of water. The analyte was eluted with 1 mL of methanol. Following solvent evaporation, the sample extract was reconstituted with 60 µL of acetonitrile: water (85:15) and 20 µL was injected to LC-MS/MS for analysis. The LC-MS/MS was performed on TSQ Quantum Access MAX triple stage quadruple mass spectrometer, coupled with Accela 1250 pump and Accela Open Autosampler (Thermo Fisher Scientific, San Jose, CA, USA). Chromatographic separation was achieved on a 150 mm x 2.1mm Altima HP HILIC column (Grace Davison Discovery Sciences, Deerfield, IL, USA) with 15% mobile phase A (water containing 0.5% acetic acid and 0.025% trifluoroacetic acid) and 85% mobile phase B (acetonitrile containing 0.5% acetic acid and 0.025% trifluoroacetic acid) at a flow rate of 0.25 ml/min. The mass spectrometer was operated in the positive ESI mode. The capillary temperature and vaporizer temperature were at 270˚C and 350˚C, respectively. The ion spray voltage was set to 3500. Nitrogen sheath and auxiliary gases were set to 35 and 10 arbitrary units, respectively. The ion pairs of m/z 284/168 and m/z 289/173 were used to monitor 8-OHdG and ^15^N_5_-8-OHdG, respectively.

*Lung morphometric analysis*

The whole lung plus trachea was removed from the chest, the right lung lobes were dissected and snap-frozen in liquid nitrogen for later analysis. Through the endotracheal catheter which was connected to a PE90 polyethylene tubing and a syringe containing 4% paraformaldehyde, the left lung was inflated and maintained with 25 cm of water pressure for at least 4 hours and then embedded in paraffin. Paraffin blocks were sectioned to expose the maximum surface area of lung tissue in the plane of the bronchial tree. Five µm sections were cut and stained with haematoxylin and eosin (H&E) or Masson’s trichrome for morphometric analysis. All counts on histology sections were performed by two investigators who were unaware of the treatment protocol of the mouse sections.

The severity of inflammatory response in peribronchial and perivascular in H&E sections was scored on a 0–3 scale: 0= no inflammatory response; 1=mild inflammation with foci of inflammatory cells in bronchial or vascular wall and in alveolar septa; 2=moderate inflammation with patchy inflammation or localized inflammation in walls of bronchi or blood vessels and alveolar septa, and less than one-third of lung cross-sectional area is involved; and 3=severe inflammation with diffuse inflammatory cells in walls of bronchi or blood vessels and alveoli septa; between one-third and two-thirds of the lung area is involved.

The mean linear intercept (L_m_), a measure of interalveolar septal wall distance, was determined using a reticule with a Thurlbeck grid comprising of 5 lines (each 550 μM long), with 10 fields per section assessed at random. Two slides per mouse were coded and analyzed. Fields with airways or vessels were avoided by moving one field in any one direction. The mean linear intercept was calculated through dividing the length of the line by the number of alveolar wall and grid line interception counted.

*Point counting for airway epithelium, collagen and airway smooth muscle*

Masson’s trichrome-stained sections were point-counted to evaluate morphological changes of airway epithelium, collagen deposition and airway smooth muscle (ASM) mass in the bronchial wall. 3-4 medium sized bronchial walls in the left mouse lung were examined using 300 point graticule at ×400 magnification, avoiding parallel blood vessel region. The points overlapping the airway epithelium, collagen, and ASM were recorded. Proportions of the above morphological changes were expressed as a percentage of accumulated viable points and calculated using the formula, percentage of epithelial area or collagen area or ASM area = number of points overlying epithelium or collagen or ASM /total sum of number of points ×100.

*Immunostaining for apoptosis protease activating factor-1 (APA-1)*

Lung sections were incubated with peroxidase blocking solution (Dako, Cambridge, UK). After incubation with rabbit anti-apoptosis protease activating factor-1 (APA-1) primary antibody [AMS Biotechnology (Europe), Milton Park, Abingdon, UK], the sections were incubated with polyclonal goat anti-rabbit horseradish peroxidase (HRP)-conjugated secondary antibody (Dako) followed by incubation with diaminobenzidine (DAB) liquid and peroxide buffer (Dako). Stained antigen sites were detected as a brown product. Slides were counterstained with H&E to provide nuclear and morphological detail. Irrelevant rabbit IgG (Sigma-Aldrich, Poole, UK) was used for the primary layer as a negative control procedure. The immunostaining intensity for APA-1 in the airway epithelium where it was mostly observed was semiquantitatively given a score ranging from 0 to 3 (0= negative, 1=weak staining, 2= moderate staining, and 3=strong staining). The number of APA-1 positive cell per field (10 fields/per mouse) was also counted under microscope (400×magnification).

*Reverse transcription and real-time PCR*

Total RNA was extracted from frozen stored lung tissue using an RNeasy Mini Kit(Qiagen, West Sussex, UK). Half of a microgram per sample of RNA was reverse transcribed using QuantiTect Rev. Transcription Kit (Qiagen). The cDNA generated was used as template in real-time PCR analyses to determine mRNA levels by using Rotor-Gene 3000 (Corbett Research, Sydney, Australia) and QuantiTect SYBR Green PCR Master Mix Reagent (Qiagen).

Sequences of primers were designed using Primer 3 software online from Simgene and synthesized by Invitrogen (Paisley,UK). The primer specificity was assessed by using the BLAST software. Melting curve analysis was carried out to ensure the presence of one specific PCR product. The sequences of the gene specific primer sets were:

IL-1β F: CAGGCAGGCAGTATCACTCA, R: ATGAGTCACAGAGGATGGGC,

MMP-9 F: CCCGCTGTATAGCTACCTCG, R: CTGTGGTTCAGTTGTGGTGG,

TGF-β F: CTGTCCAAACTAAGGCTCGC, R: AGCATAGTAGTCCGCTTCGG,

Caspase-3 F: AGTCTGACTGGAAAGCCGAA, R: AGCCTCCACCGGTATCTTCT,

SOD2 F: CCAAAGGAGAGTTGCTGGAG, R: GAACCTTGGACTCCCACAGA,

HO-1 F: TGCTCGAATGAACACTCTGG, R: TCCTCTGTCAGCATCACCTG,

18s F: CTTAGAGGGACAAGTGGCG, R:ACGCTGAGCCAGTCAGTGTA,

PCR conditions were as follows: initial heat activation, 15 min at 95°C; denaturation, 15 s at 95°C; annealing, 20 s at 61°C; extension, 20 s at 72°C; 45 cycles.

Results from the reaction were analyzed using a standard curve and Rotor-Gene6 software (Corbett Research). Relative abundance of gene expression was normalized to 18S rRNA expression.

**Legend to Figure S1.**

Protocol used in the six experimental groups.

Reference List

(S1) Triantaphyllopoulos K, Hussain F, Pinart M, et al. A model of chronic inflammation and pulmonary emphysema after multiple ozone exposures in mice. *Am J Physiol Lung Cell Mol Physiol* 2011 May,**300**(5), L691-L700.

(S2) Vanoirbeek JA, Rinaldi M, De V, V, et al. Noninvasive and invasive pulmonary function in mouse models of obstructive and restrictive respiratory diseases. *Am J Respir Cell Mol Biol* 2010 Jan,**42**(1), 96-104.

(S3) Williams AS, Leung SY, Nath P, et al. Role of TLR2, TLR4, and MyD88 in murine ozone-induced airway hyperresponsiveness and neutrophilia. *J Appl Physiol* 2007 Oct,**103**(4), 1189-1195.

(S4) Williams AS, Nath P, Leung SY, et al. Modulation of ozone-induced airway hyperresponsiveness and inflammation by interleukin-13. *Eur Respir J* 2008 Sep,**32**(3), 571-578.
